# Enhanced Detection of Antigen-Specific CD4^+^ T Cells Using Altered Peptide Flanking Residue Peptide–MHC Class II Multimers

**DOI:** 10.4049/jimmunol.1402787

**Published:** 2015-11-09

**Authors:** Christopher J. Holland, Garry Dolton, Martin Scurr, Kristin Ladell, Andrea J. Schauenburg, Kelly Miners, Florian Madura, Andrew K. Sewell, David A. Price, David K. Cole, Andrew J. Godkin

**Affiliations:** * Division of Infection and Immunity, Cardiff University School of Medicine, Cardiff CF14 4XN, Wales, United Kingdom; and; † Department of Integrated Medicine, University Hospital of Wales, Cardiff CF14 4XW, Wales, United Kingdom

## Abstract

Fluorochrome-conjugated peptide–MHC (pMHC) class I multimers are staple components of the immunologist’s toolbox, enabling reliable quantification and analysis of Ag-specific CD8^+^ T cells irrespective of functional outputs. In contrast, widespread use of the equivalent pMHC class II (pMHC-II) reagents has been hindered by intrinsically weaker TCR affinities for pMHC-II, a lack of cooperative binding between the TCR and CD4 coreceptor, and a low frequency of Ag-specific CD4^+^ T cell populations in the peripheral blood. In this study, we show that peptide flanking regions, extending beyond the central nonamer core of MHC-II–bound peptides, can enhance TCR–pMHC-II binding and T cell activation without loss of specificity. Consistent with these findings, pMHC-II multimers incorporating peptide flanking residue modifications proved superior for the ex vivo detection, characterization, and manipulation of Ag-specific CD4^+^ T cells, highlighting an unappreciated feature of TCR–pMHC-II interactions.

## Introduction

The key molecular event that governs adaptive cellular immunity is αβ TCR binding to peptide–MHC (pMHC) (1). In contrast to Abs, which bind diverse antigenic structures with high affinities (*K*_D_ values in the nanomolar to picomolar range), TCR binding is characterized by weak affinities (*K*_D_ > 1 μM) and fast dissociation rates at physiological temperatures (2, 3). Accordingly, soluble pMHC molecules must be multimerized to bind stably on the surface of Ag-specific T cells via a slower composite dissociation rate (half-life in the range of minutes to hours) compared with the equivalent monomeric TCR–pMHC interaction (half-life of seconds) (4, 5). Fluorochrome-tagged versions of such pMHC multimers enable the visualization of Ag-specific T cells, often becoming internalized to generate a durable intracellular label (6).

Since their development (4), pMHC class I (pMHC-I) multimers have revolutionized studies of Ag-specific CD8^+^ T cells (7). However, pMHC class II (pMHC-II) multimer technology for the analysis of Ag-specific CD4^+^ T cells has progressed at a much lower rate. Three key issues have affected the wider use of pMHC-II multimers: 1) methodological problems with the production of recombinant pMHC-II heterodimers; 2) typically low frequencies of peripheral Ag-specific CD4^+^ T cells (4, 8–10); and 3) weak pMHC-II multimer binding at the cell surface (11–15). It is notable in this regard that TCR binding affinities for pMHC-II are approximately five times weaker compared with their pMHC-I counterparts (2), a distinction that could be linked to differences in Lck delivery by the CD4 and CD8 coreceptors (16). Furthermore, the CD8 coreceptor interaction with pMHC-I, which occurs at *K*_D_ of ∼100–200 μM (17–19), stabilizes the TCR–pMHC-I interaction (20) and thereby enhances pMHC-I multimer binding avidity (20–23). In contrast, the CD4 coreceptor binds pMHC-II with an estimated *K*_D_ of ∼200 μM to >2 mM (24), which is likely insufficient to stabilize TCR–pMHC-II interactions at the cell surface (25, 26).

In humans, the most abundantly expressed MHC-II element on blood-derived APCs is the HLA-DR αβ heterodimer. The core epitope is a nonamer peptide bound through a series of pockets in the HLA-DR molecule at positions (P)1, P4, P6, and P9 (27). However, the binding groove of the HLA-DR molecule is open at either end, allowing longer peptides to bind with the nonbound peptide flanking regions (PFRs) extending from the N terminus (e.g., P^−^2 and P^−^1) and C terminus (e.g., P10 and P11) (28, 29). Indeed, longer PFRs have been shown to increase peptide binding affinity for MHC-II (30, 31). In a previous study, we eluted and sequenced multiple ligands from a range of MHC-II molecules and identified enrichment patterns in the nonbound PFRs, particularly basic residues at the C terminus, reflecting preferential Ag processing mechanisms (32, 33). Moreover, basic residue substitutions in the C-terminal PFR of known T cell epitopes from a wide range of Ags, including hemagglutinin (HA), HIV-1 Gag, myelin basic protein, tetanus toxoid, and hen egg lysozyme, can lead to increased CD4^+^ T cell responses (33–35). Several mechanisms could potentially account for the increased immunogenicity of MHC-II–restricted T cell epitopes with such “basic tails,” including 1) enhanced processing by APCs, 2) increased peptide binding to MHC-II and 3) improved TCR binding to pMHC-II. We recently used surface plasmon resonance (SPR) to show that substitutions in the C-terminal PFR of an influenza HA epitope could increase pMHC-II binding affinity for a wide range of different TCRs (35). In the present study, we examine whether this effect can be harnessed to improve the detection and characterization of Ag-specific CD4^+^ T cells directly ex vivo.

## Materials and Methods

### Peptides

All peptides were synthesized commercially at >80% purity (EMC Microcollections). The following peptides were used in this study: 1) influenza HA_306–318_ (Flu1), PKYVKQNTLKLAT (core epitope positions P1–P9 underlined), 2) C-terminally modified variants of HA_306−318_ with Arg at P11 (Flu3, PKYVKQNTLKLA**R**); and 3) HIV-1 Gag p24_299–312_, DRFYKTLRAEQASQ.

### Cell culture

Healthy donors were HLA genotyped using allele-specific PCRs (Welsh Blood Transfusion Service). PBMCs were isolated from DR1^+^ donors by density gradient centrifugation of heparinized blood, layered onto an equal volume of LymphoPrep (Axis-Shield). All human samples were used in accordance with United Kingdom guidelines. T cell lines and clones were generated and maintained as described previously (36). Ag-specific T cell clones 2C5, DC08, and DCD10 were derived either by limiting dilution or by DR1-HA_306−318_ multimer staining and cell sorting. Clonality was confirmed by sequencing *TRA* and *TRB* gene rearrangements. HLA restriction was confirmed via peptide titrations in the presence of DR1-matched and DR1-mismatched B lymphoblastoid cell lines (B-LCLs) (37).

### Generation of expression plasmids

The HA1.7 and 2C5 TCRs were generated from the corresponding CD4^+^ T cell clones as described previously (38). All sequences were confirmed by automated DNA sequencing (Lark Technologies). A disulfide-linked construct was used to produce the soluble domains (variable and constant) for both the TCRα- and TCRβ-chains (39). The DRα1*0101 chain, tagged with a biotinylation sequence, and DRβ1*0101 chain were also cloned and used to produce the pMHC-II molecules (40). All constructs were inserted into separate pGMT7 expression plasmids under the control of the T7 promoter.

### Protein expression and purification

Rosetta DE3 *Escherichia coli* competent cells were used to express the TCRα, TCRβ, DRα1*0101, and DRβ1*0101 chains in the form of inclusion bodies as described previously (38). The DRα1*0101 and DRβ1*0101 chains were purified into 8 M urea buffer (8 M urea, 20 mM Tris [pH 8.1], 0.5 mM EDTA, 30 mM DTT) by ion exchange using a HiTrap Q column (GE Healthcare, Buckinghamshire, U.K) to remove bacterial impurities. For a 1-l TCR refold, 30 mg TCRα-chain was incubated for 15 min at 37°C with 10 mM DTT and added to cold refold buffer (50 mM Tris [pH 8.1], 2 mM EDTA, 2.5 M urea, 6 mM cysteamine hydrochloride, 4 mM cystamine). After 15 min, 30 mg TCRβ-chain, also incubated for 15 min at 37°C with 10 mM DTT, was added. TCR refolds were dialyzed against 20 vol 10 mM Tris and purified by ion exchange (Poros 50 HQ, Life Technologies, Cheshire, U.K.) and gel filtration (S200 HR, GE Healthcare). For a 1-l pMHC-II refold, 2 mg DRα1*0101 chain was mixed with 2 mg DRβ1*0101 chain and 0.5 mg peptide for 15 min at 37°C with 10 mM DTT (41). This mixture was then added dropwise to cold refold buffer (25% glycerol, 20 mM Tris [pH 8.1], 1 mM EDTA, 2 mM glutathione reduced, 0.2 mM glutathione oxidized). Refolds were incubated for 72 h at 4°C. Subsequently, the refold mixture was concentrated using a VivaFlow 200 (polyethersulfone membrane) module (Sartorius) to a volume of ≤50 ml and then further concentrated to a volume of ≤2 ml using a Vivaspin 20 (Sartorius). Concentrated refolds were washed with PBS. The refold was then purified by affinity chromatography by binding to a conformationally specific Ab (L243) and size exclusion chromatography using a Superdex 200 HR column and an ÄKTA FPLC in conjunction with Unicorn software. Protein quality was analyzed by Coomassie-stained SDS-PAGE. Biotinylated pMHC-II was prepared as described previously (42).

### Surface plasmon resonance

Binding analysis was performed using a Biacore 3000 (GE Healthcare) equipped with a CM5 sensor chip, as reported previously (43, 44). Between 200 and 400 response units of biotinylated pMHC-II was immobilized on streptavidin, which was chemically linked to the chip surface. pMHC-II was injected at a slow-flow rate (10 μl/min) to ensure uniform distribution on the chip surface. The 2C5 TCR was purified and concentrated to ∼100 μM on the day of analysis to minimize TCR aggregation. For equilibrium analysis, eight serial dilutions were carefully prepared and injected over the relevant sensor chip at a flow rate of 45 μl/min at 25°C. Results were analyzed using BIAevaluation 3.1, Microsoft Excel, and Origin 6.1. The *K*_D_ values were calculated using a nonlinear curve fit [*y* = P_1_*x*/(P_2_ + *x*)].

### Thermodynamic analysis

Thermodynamic analysis of HA1.7 TCR ligation to DR1-Flu3 was performed using a Biacore T100 (GE Healthcare) equipped with a CM5 sensor chip. Activation and pMHC-II loading were assessed as previously described (40). A total of 500 response units of biotinylated pMHC-II were immobilized to streptavidin, which was chemically linked to the chip surface. Equilibrium binding analysis was performed with 10 serial dilutions of the TCR individually injected at temperatures of 5, 10, 15, 20, 25, and 30°C. Experiments were performed in triplicate using a blank flow cell, A2-ELAGIGILTV and DR1-CLIP as negative controls in separate experiments. The binding response was determined by subtraction of the response measured on a negative control flow cell from the response measured on flow cells containing DR1-Flu3. Results were analyzed using BIAevaluation 3.1, Microsoft Excel, and Origin 6.1. The *K*_D_ values were calculated using a nonlinear curve fit [*y* = (P_1_*x*)/(P_2_ + *x*)]. Thermodynamic parameters were calculated using the nonlinear van’t Hoff equation [RT ln *K*_D_ = ΔH° − TΔS° + ΔCp°(T − T_0_) − TΔCp° ln (T/T_0_)], where R is the gas constant, T is the temperature in Kelvin (K) at which the *K*_D_ is measured, T_0_ is the reference temperature (298.15 K = 25°C), ΔH° is the enthalpy change upon binding at T_0_ (cal⋅mol^−1^), ΔS° is the standard entropy change upon binding at T_0_ (cal⋅mol^−1^⋅K^−1^), and ΔCp° is the specific heat capacity (cal⋅mol^−1^⋅K^−1^).

### pMHC-II multimer staining of CD4^+^ T cell clones

Clonal CD4^+^ T cells (1 × 10^5^) specific for HA_306–318_ were resuspended with 1 μg DR1-Flu1 or DR1-Flu3 multimer and incubated for 20 min at 4°C (45). Cells were then washed twice with PBS, stained with Live/Dead fixable violet (ViViD; Invitrogen) for 10 min at room temperature and labeled with mouse anti-human CD4-allophycocyanin (BD Biosciences) for 20 min at 4°C. After two further washes in PBS, samples were acquired directly on a FACSCanto II flow cytometer (BD Biosciences). Data were analyzed using FlowJo software version 10 (Tree Star). All incubations with fluorochrome-conjugated pMHC-II multimers were performed in the dark.

### Multimer dilution assay

Each multimer was serially diluted six times (1, 0.5, 0.25, 0.125, 0.0625, 0.0312, or 0.0156 μg with respect to the pMHC-II component) and used in a final volume of 20 μl to stain 1 × 10^6^ DR1^+^ PBMCs spiked at 1% with the CD4^+^ T cell clone 2C5. After incubation for 20 min at 4°C, cells were washed twice in PBS, stained with Live/Dead fixable aqua (Invitrogen) for 10 min at room temperature and labeled with mouse anti-human CD3-FITC (Serotec), mouse anti-human CD4-allophycocyanin (BD Biosciences), mouse anti-human CD14–Pacific Blue (Invitrogen), and mouse anti-human CD19–Pacific Blue (Invitrogen) for 20 min at 4°C. After two further washes in PBS, samples were acquired directly on a FACSCanto II flow cytometer (BD Biosciences). Data were analyzed using FlowJo software version 10 (Tree Star).

### Clone dilution assay

PBMCs (2 × 10^6^) were spiked at 1, 0.5, 0.25, 0.125, 0.0625, or 0.0312% with the CD4^+^ T cell clone 2C5. Cell mixtures were then stained with 0.125 μg multimer/1 × 10^6^ PBMCs and labeled with Abs as described in the previous section. Samples were acquired directly on a FACSCanto II flow cytometer (BD Biosciences) and analyzed using FlowJo software version 10 (Tree Star).

### Multimer staining following PBMC culture

Flu1 peptide–stimulated, precultured PBMCs (5 × 10^5^) were transferred to a 96-well plate and washed with PBS. Cell pellets were resuspended in 25 nM phosphate kinase inhibitor dasatinib (Axon Medchem, Reston, VA) and incubated for 30 min at 37°C. For each multimer, 0.5 μg was added directly to the cell pellet before incubating for 20 min on ice in the dark. Where dual staining with both PE- and allophycocyanin-conjugated multimers was used, 0.5 μg each was mixed per sample prior to addition to the cell pellets. Cells were then washed twice with cold PBS, followed by Live/Dead aqua (Life Technologies) staining to exclude dead cells. Surface staining was subsequently performed in PBS, 2.5% FCS, and 5 mM EDTA (FACS buffer), with the following cell surface markers: CD3-PerCP-Cy5.5 (BD Biosciences), CD4–Brilliant Violet 421 (BioLegend), CD14-FITC (BioLegend), and CD19-FITC (BioLegend). Cells were incubated for 20 min at 4°C in the dark before resuspending in FACS buffer for acquisition on a FACSCanto II (BD Biosciences). Data were analyzed using FlowJo version 10 (Tree Star).

### Direct ex vivo multimer staining and phenotypic analysis of DR1^+^ PBMCs

One healthy DR1^+^ volunteer (donor 1) was inoculated with the 2011–2012 trivalent influenza vaccine, containing the immunodominant HA_306–318_ epitope. Peripheral blood (50 ml) was obtained before vaccination and on days 3, 7, 11, and 14 after vaccination, processed as described above, and stored in liquid nitrogen. Additionally, 50 ml peripheral blood was obtained from two healthy unvaccinated DR1^+^ volunteers (donors 2 and 3). Frozen PBMCs were thawed and washed twice with multimer buffer (PBS, 1% FCS). PBMCs were treated with 25 nM protein kinase inhibitor dasatinib for 60 min at 37°C. PBMCs were then stained with 0.5 μg pMHC-II multimer/1–4 × 10^6^ PBMCs for 30 min at 4°C, and washed with ice cold PBS. PBMCs were then subsequently stained with ViViD Live⁄Dead cell stain (Invitogen) for 10 min at room temperature followed by a costaining Ab mixture that included mouse anti-human CD3 allophycocyanin-H7 (BD Biosciences), mouse anti-human CD4-PE-Cy5.5 (Life Technologies), mouse anti-human CD8-BV711 (BioLegend), mouse anti-human CD14–Pacific Blue (Invitrogen), mouse anti-human CD19–Pacific Blue (Invitrogen), mouse anti-human CD27-PE-Cy5 (Beckman Coulter), mouse anti-human CD45RO-ECD (Beckman Coulter), and mouse anti-human CD57-FITC (BD Biosciences) and incubated at 4°C for 20 min. Alternatively, cocktails contained mouse anti-human CD3-PerCP (Miltenyi Biotec), mouse anti-human CD4-FITC (Miltenyi Biotec), mouse anti-human CD8-PE (Miltenyi Biotec), mouse anti-human CD14–Pacific Blue (BioLegend), and mouse anti-human CD19–Pacific Blue (BioLegend). Samples from donors 2 and 3 were treated with pMHC multimer stabilizing anti-fluorochrome Abs as described in Tungatt et al. (46). Samples were washed with multimer buffer, resuspended in PBS, directly acquired on a FACSAria II (BD Biosciences), and analyzed using FlowJo version 10 (Tree Star). The percentage shown indicates the proportion of CD3^+^ T cells stained with each multimer reagent.

### Direct cloning of CD4^+^DR1-Flu3^+^ T cells sorted with modified pMHC-II multimers

Frozen PBMCs from day 7 postvaccination were stained as described in the previous section. CD4^+^multimer^+^ cells were then sorted by flow cytometry at 1 cell/well into 96-well round-bottom plates containing 5 × 10^4^ irradiated allogeneic mixed PBMCs from three donors and 1 μl/ml PHA in 100 μl CD4^+^ T cell medium comprising RPMI 1640 (Life Technologies), 10% FCS (Sigma-Aldrich), 1% heat-inactivated human AB serum (Welsh Blood Transfusion Service), 0.02 M HEPES (Sigma-Aldrich), 2 mM l-glutamine (Life Technologies), 1 mM nonessential amino acids (Life Technologies), 1 mM sodium pyruvate (Life Technologies), 100 IU/ml penicillin (Life Technologies), 100 μg/ml streptomycin (Life Technologies), and 20 IU/ml human rIL-2 (Proleukin; University Hospital of Wales Pharmacy). Clonal expansion plates were incubated for 7 d at 37°C, 5% CO_2_. Wells were then replenished with 100 μl CD4^+^ T cell medium and incubated for a further 7 d at 37°C, 5% CO_2_. Clones were analyzed for Ag specificity via IFN-γ ELISA.

### IFN-γ ELISA

Clonal CD4^+^ T cells (10,000 per triplicate test well) were incubated overnight with 5 × 10^4^ DR1^+^ B-LCLs in the presence or absence of 10 μg/ml peptide (Flu1 or Flu3). IFN-γ release was quantified by ELISA according to the manufacturer’s instructions (R&D Systems).

## Results

### Improved TCR affinity binding to C-terminal PFR-modified peptides

**FIGURE 1.**
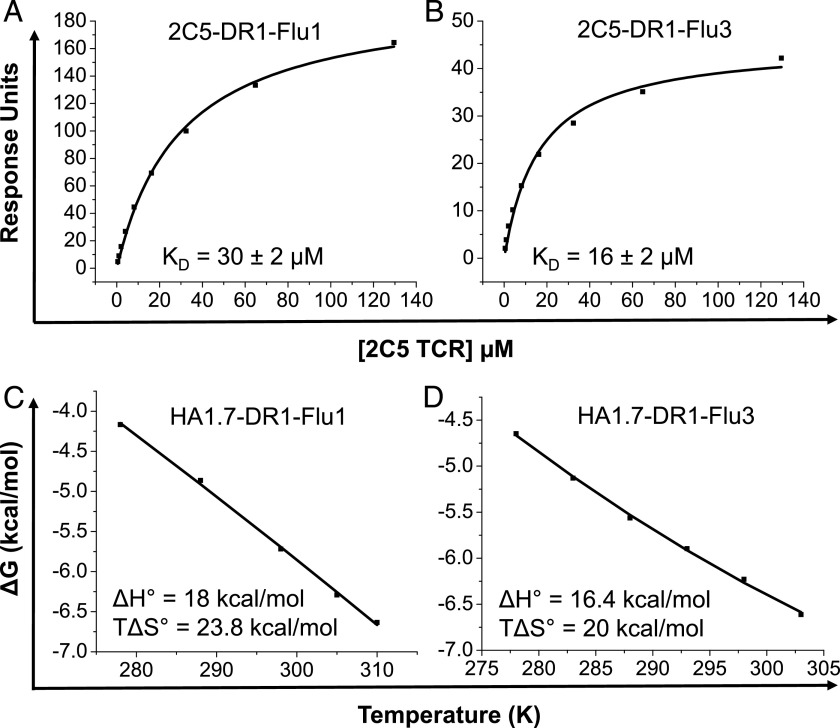
The biophysical impact of altered C-terminal PFRs on TCR binding. SPR was used to determine equilibrium binding of the 2C5 TCR to DR1 complexed with either (**A**) native Flu1 (HA_306−318_, PKYVKQNTLKLAT) or (**B**) altered Flu3 (PKYVKQNTLKLAR). (**C**) Previously published thermodynamic analysis of the HA1.7-DR1-Flu1 interaction (40). (**D**) HA1.7-DR1-Flu3 thermodynamic properties showing enthalpy (ΔH^o^) and entropy (TΔS^o^) at 298 K calculated by nonlinear regression of temperature (K) plotted against free energy (ΔG^o^).

Our previously reported biophysical data demonstrate that targeted substitutions in the C-terminal PFRs of MHC-II epitopes can substantially enhance TCR binding affinity (35). To extend these preliminary results and confirm the generality of this observation, we derived a new DR1-restricted CD4^+^ T cell clone (2C5) specific for the HA_306−318_ epitope (Flu1, PKYVKQNTLKLAT; core binding P1–P9 nonamer underlined). The 2C5 TCR was subsequently cloned, expressed, and refolded as a soluble protein for biophysical analyses. In SPR experiments, the 2C5 TCR bound DR1-Flu3 (PKYVKQNTLKLA**R**) with an affinity ∼2-fold stronger compared with the corresponding wild-type C-terminal PFR sequence (Fig. 1A, 1B). This finding was reproduced with three other DR1-restricted TCRs specific for HA_305−320_ (HA1.7, F11, and C6) where binding affinity increased by ∼1.4- to 1.8-fold with Arg substituted at P10 (Flu2, PKYVKQNTLKL**R**T) and by ∼2-fold with Arg at P11 (Flu3, PKYVKQNTLKLA**R**) (35). These differences in binding affinity were further verified using a DR1-restricted TCR specific for an HIV-1 Gag-derived peptide epitope (data not shown). Modifications to C-terminal PFRs can therefore enhance TCR binding affinity across multiple clonotypes and specificities, implying a general mechanism for TCR interactions with MHC-II–presented PFRs.

In an earlier report, we explored the thermodynamics of the HA1.7 TCR interaction with DR1-Flu1, demonstrating that a net decrease in electrostatic interactions (unfavorable enthalpy, ΔH^o^ = 18 kcal/mol) and an energetically favorable transition from order to disorder probably mediated the binding energy of the interaction (favorable entropy, TΔS^o^ = 23.8 kcal/mol) (40). To extend these findings, we performed a thermodynamic analysis of HA1.7 TCR binding to both DR1-Flu1 and DR1-Flu3 (Fig. 1C, 1D). These experiments revealed a change in enthalpy (ΔH^o^) toward a more favorable state for the interaction with DR1-Flu3 (18 → 16.4 kcal/mol). As predicted previously by structural modeling (35), these data suggest an increase in net electrostatic interactions compared with HA1.7-DR1-Flu1 and offer a thermodynamic explanation for increased affinity TCR binding to an HLA-DR–presented peptide with a substituted PFR.

### C-terminal PFR modifications enhance DR1 multimer staining

**FIGURE 2.**
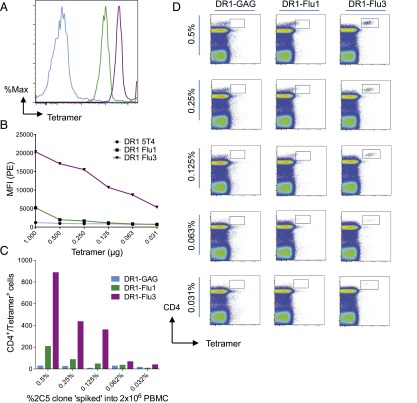
pMHC-II multimers with modified PFRs enable the detection of low-frequency Ag-specific CD4^+^ T cell clones. Arg substitution at P11 of the HA_306−318_ epitope (Flu3) substantially increases pMHC-II multimer staining. (**A**) Histograms showing negative control (blue), DR1-Flu1 (green), and DR1-Flu3 (purple) multimer staining of the 2C5 clone. (**B**) Graph showing staining MFIs generated with different multimers as indicated across a range of concentrations. (**C**) Bar graph showing the number of CD4^+^multimer^+^ cells detected with the indicated multimers plotted against the frequency (%) of clonal 2C5 cells spiked into PBMCs. (**D**) Representative flow cytometry plots showing staining with the indicated multimers, at a concentration of 0.125 μg/1 × 10^6^ PBMCs, across a range of 2C5 “spiked” frequencies (%) in PBMCs. The gating strategy is shown in Supplemental Fig. 1.

To determine whether increased TCR binding affinity could enhance the detection of CD4^+^ T cells using pMHC-II multimers, we generated fluorochrome-tagged DR1-Flu1 and DR1-Flu3 multimers. A large increase in mean fluorescence intensity (MFI) was observed with the DR1-Flu3 multimer versus the DR1-Flu1 multimer when staining the 2C5 clone (Fig. 2A, gating strategy shown in Supplemental Fig. 1). Similar marked shifts in MFI were observed with other CD4^+^ T cell clones specific for HA_305−320_ (data not shown). These results demonstrate that relatively small differences in monomeric TCR binding affinity (∼2-fold) can substantially enhance multimer binding avidity at the cell surface.

The superior binding of DR1-Flu3 multimers was confirmed in two further head-to-head comparisons with DR1-Flu1 multimers: 1) reagent titrations, and 2) cognate CD4^+^ T cell titrations. Initially, 1 × 10^6^ DR1^+^ PBMCs were spiked at 1% with a Flu1-specific CD4^+^ T cell clone. Although both multimers identified a CD4^+^multimer^+^ population when used at a concentration of 1 μg/1 × 10^6^ PBMCs, only the enhanced staining intensity observed with the DR1-Flu3 multimer enabled clear visualization and accurate enumeration (Fig. 2B). At lower reagent concentrations, the DR1-Flu1 multimer failed to identify cognate CD4^+^ T cells. In contrast, distinct staining was apparent with the DR1-Flu3 multimer at concentrations down to 0.0312 μg/1 × 10^6^ PBMCs. Unless stated otherwise, all multimer stains were performed using 0.125 μg/1 × 10^6^ PBMCs. This represents the optimal quantity of DR1-Flu3 multimer per stain that produced the greatest shift in MFI while reducing potential nonspecific staining. Subsequently, the frequency of spiked Flu1-specific CD4^+^ T cells was titrated to determine any differences in detection sensitivity (Fig. 2C, 2D). The DR1-Flu3 multimer displayed a far superior ability to detect cognate CD4^+^ T cells across the entire range of spiked frequencies compared with the DR1-Flu1 multimer. Indeed, precise quantification of target CD4^+^ T cells with the DR1-Flu1 multimer was challenging due to poor separation and almost impossible at frequencies ≤0.25%.

**FIGURE 3.**
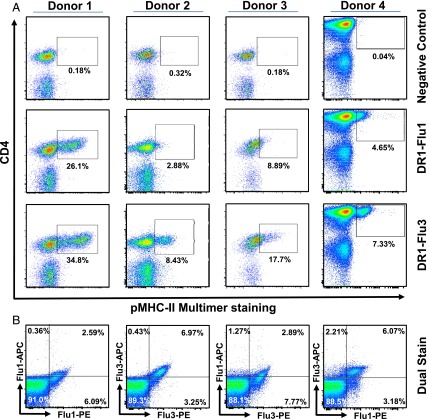
pMHC-II multimers with modified PFRs enable enhanced detection of expanded Ag-specific CD4^+^ T cells. PBMCs from 4 DR1^+^ donors were cultured with the HA_306−318_ (Flu1) peptide for 12 d. DR1-Gag multimers were used as a negative control. (**A**) Representative flow cytometry plots showing DR1-Flu1, or DR1-Flu3 multimer staining of DR1^+^ PBMCs cultured for 12 d. (**B**) Dual staining performed on donor 4 using DR1-Flu1-PE/DR1-Flu1-allophycocyanin, DR1-Flu3-PE/DR1-Flu3-allophycocyanin, DR1-Flu1-PE/DR1-Flu3-allophycocyanin, or DR1-Flu3-PE/DR1-Flu1-allophycocyanin multimer mixes.

In further experiments, we stained PBMCs from four healthy DR1^+^ volunteers 12 d after HA_306–318_ peptide stimulation (Fig. 3A). The DR1-Flu3 multimer detected larger populations of CD4^+^multimer^+^ cells, with generally greater MFIs, compared with the DR1-Flu1 multimer in all cases. These numerical differences suggest that the DR1-Flu3 multimer can detect lower avidity cognate CD4^+^ T cells that remain unstained with the DR1-Flu1 multimer (15). We also performed dual staining experiments in which the DR1-Flu1 and DR1-Flu3 multimers were conjugated to different fluorochromes (DR1-Flu1-PE/DR1-Flu3-allophycocyanin and vice versa) to determine the level of overlap between cell populations detected with each reagent (Fig. 3B). This analysis demonstrated a very similar pattern of staining compared with controls in which a single reagent was used with two different fluorochromes (i.e., DR1-Flu1-PE/DR1-Flu1-allophycocyanin and DR1-Flu3-PE/DR1-Flu3-allophycocyanin), suggesting that most CD4^+^ T cells were detected by both reagents. In all cases, the PE reagents were brighter than the allophycocyanin reagents, generating a degree of bias in the analysis. However, a comparison between DR1-Flu1-PE from dual stain 1 and DR1-Flu3-PE from dual stain 2, or DR1-Flu1-allophycocyanin from dual stain 1 and DR1-Flu3-allophycocyanin from dual stain 2, suggested that DR1-Flu3 was able to detect additional, presumably lower avidity, cells compared with DR1-Flu1.

### Increased avidity of DR1-Flu3 multimers facilitates the ex vivo characterization and isolation of cognate T cells

**FIGURE 4.**
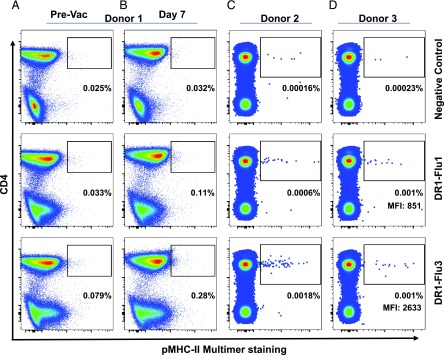
Direct ex vivo PFR-modified pMHC-II multimer staining of CD4^+^ T cells specific for HA_306–318_. Flow cytometry plots showing DR1-Flu1 and DR1-Flu3 multimer staining of PBMCs isolated ex vivo from three donors. (**A**) Donor 1 prevaccination, (**B**) donor 1 at day 7 postvaccination with the 2011–2012 trivalent influenza vaccine, (**C**) donor 2 without vaccination, and (**D**) donor 3 without vaccination. DR1-Gag and/or DR4-CLIP multimers were used as a negative control. Multimer^+^ gate cut-offs were determined from the relevant fluorescence minus one (FMO) controls (Supplemental Fig. 2). The percentage shown indicates the proportion of CD3^+^ T cells stained with each tetramer reagent.

Antiviral immune responses typically generate large numbers of Ag-specific CD8^+^ T cells that are easy to detect ex vivo using pMHC-I multimers (47, 48). In contrast, the direct ex vivo visualization and characterization of Ag-specific CD4^+^ T cells has proved problematic, often relying on additional manipulations such as magnetic bead enrichment or sampling from compartments with high precursor frequencies (8, 49, 50). To determine whether DR1-Flu3 multimers could be used to stain CD4^+^ T cells directly ex vivo, PBMCs were obtained from one healthy DR1^+^ volunteer before and after immunization with the 2011–2012 trivalent seasonal influenza vaccine, which incorporates the HA_306–318_ epitope, and in two further DR1^+^ volunteers that were not vaccinated. All three donors displayed robust T cell responses to HA_306–318_ in IFN-γ ELISPOT assays (data not shown). Direct ex vivo multimer analysis was performed using unmanipulated PBMCs obtained prevaccination (day 0) and at day 7 postvaccination in one donor, and without vaccination in a further two donors (Fig. 4). All gate “cut-offs” determined from relevant FMOs are shown in Supplemental Fig. 2. In donor 1 at days 0 and 7, and in donor 2, the DR1-Flu3 multimer stained a higher proportion of CD4^+^ T cells than did the DR1-Flu1 multimer. In donor 3, although the proportion of CD4^+^ T cells detected with each reagent was similar, the DR1-Flu3 multimer was substantially brighter, evidenced by a 3-fold higher MFI.

**FIGURE 5.**
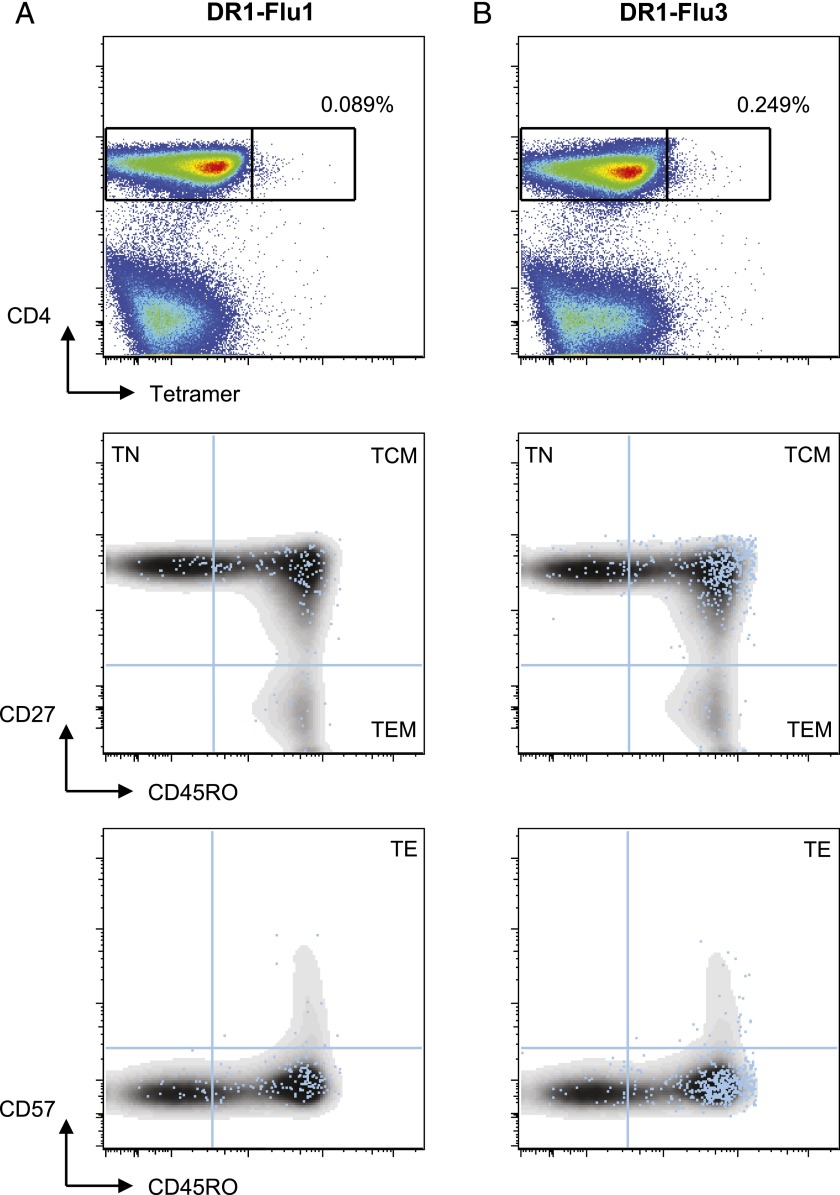
Phenotypic analysis of PBMCs stained with PFR-modified multimers after immunization with trivalent influenza vaccine. *Top panels*, Flow cytometry plots showing (**A**) DR1-Flu1 and (**B**) DR1-Flu3 multimer staining of PBMCs from a representative donor at day 7 postvaccination with the 2011–2012 trivalent influenza vaccine. *Middle panels*, Bivariate flow cytometry plots showing CD27 versus CD45RO expression on (A) DR1-Flu1 and (B) DR1-Flu3 multimer^+^ (blue dots) and multimer^−^ (density plots) cells. *Bottom panels*, Bivariate flow cytometry plots showing CD45RO versus CD57 expression on (A) DR1-Flu1 and (B) DR1-Flu3 multimer^+^ (blue dots) and multimer^-^ (density plots) cells. Multimer^+^ gate cut-offs were determined from the relevant fluorescence minus one (FMO) controls.

To investigate the true nature of these low-frequency CD4^+^multimer^+^ T cell populations, we performed a comprehensive phenotypic analysis of cellular differentiation and maturity. Representative flow cytometry plots at day 7 postvaccination are shown in Fig. 5. As previously shown, the DR1-Flu3 multimer identified a larger population of CD4^+^ T cells compared with the DR1-Flu1 multimer, enabling a more robust analysis of surface marker expression. The CD4^+^multimer^+^ populations contained a greater percentage of central memory (CD27^+^CD45RO^+^) cells (>80%) and a lower percentage of naive (CD27^+^ CD45RO^-^) cells compared with the CD4^+^multimer^−^ populations. Most cells in both the multimer^+^ and multimer^−^ compartments were CD57^−^, consistent with a low incidence of CD4^+^ T cell senescence (51).

**FIGURE 6.**
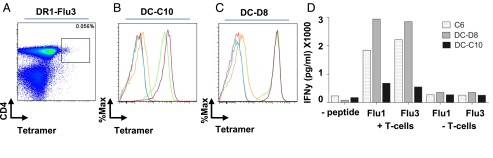
Specificity of CD4^+^ T cells isolated with PFR-modified pMHC-II multimers. (**A**) Flow cytometry plot showing DR1-Flu3 multimer staining of PBMCs from a representative donor at day 7 postvaccination with the 2011–2012 trivalent influenza vaccine. (**B** and **C**) Histograms showing multimer staining of the CD4^+^ T cell clones (B) DC-C10 and (C) DC-D8 color coded as follows: multimer FMO (red), A2-ELA multimer (negative control, blue), DR1-Gag multimer (negative control, orange), DR1-Flu1 multimer (green), and DR1-Flu3 multimer (purple). (**D**) Bar graph showing IFN-γ release by the CD4^+^ T cell clones C6, DC-C10, and DC-D8 under the indicated conditions determined via ELISA.

To confirm that the DR1-Flu3 multimer identified a population of CD4^+^ T cells specific for the HA_306–318_ epitope, we sorted and cloned multimer^+^ cells directly from unmanipulated PBMCs (Fig. 6A). Two clones (DC-C10 and DC-D8) were generated using this procedure, each of which stained with the DR1-Flu1 and DR1-Flu3 multimers (Fig. 6B, 6C). Moreover, both clones released IFN-γ after stimulation with cognate peptide–pulsed DR1^+^ B-LCLs (Fig. 6D).

## Discussion

Soluble fluorochrome-conjugated multimeric pMHC-I molecules have revolutionized the field of cellular immunology by enabling the direct ex vivo visualization, enumeration, characterization, and isolation of Ag-specific CD8^+^ T cell populations (4, 7). In contrast, technical and biological constraints have hindered the development of pMHC-II multimers for the ex vivo study of Ag-specific CD4^+^ T cells (4, 8–10). As a consequence, detailed analyses of CD4^+^ T cell function in humans have relied on laborious enrichment procedures (7, 45, 52–54) and/or studies restricted to anatomical compartments more directly affected by the immune response of interest (49, 55). However, such approaches are not universally successful (11–14), most likely due to weaker TCR binding affinities and a lack of CD4 coreceptor stabilization. Cumulatively, these factors negatively impact the binding avidity of pMHC-II multimers and thereby intrinsically limit the utility of these reagents for the comprehensive identification of functional Ag-specific CD4^+^ T cells.

Although the MHC-I and MHC-II peptide-binding grooves are very similar in overall conformation, both comprising two “sides” formed from α helices and a floor of β sheets, the nature of the bound peptide in each MHC class is distinct. For example, the MHC-I binding groove is “closed” at both ends, with two main anchors at the N and C terminus of the peptide (56). This conformation generally forces longer peptides to bulge in the center to accommodate extra residues (57). In contrast, the MHC-II peptide groove is open at each end, enabling the core 9-mer peptides to bind in a stretched conformation and extend beyond the limits of groove, forming PFRs (27). This presentation mode is mediated by MHC-II binding pockets that interact with peptide positions P1, P4, P6, and P9 via a network of electrostatic interactions. Modification of this network has been shown to directly alter pMHC-II stability, demonstrating the importance of these interactions for the stable display of epitopes at the cell surface for T cell interrogation (58, 59). Peptide modifications at these key binding sites, as well as in other positions along the 9-mer core, have also been shown to directly affect the sensitivity of CD4^+^ T cells (60–64). Included in these studies are multiple examples of single peptide substitutions in the core 9-mer region of the epitope that select different T cell specificities, demonstrated by T cell activation and/or tetramer binding (60, 61). Intriguingly, the open nature of the MHC-II binding groove also enables the possibility of multiple peptide binding registers. Similarly to single peptide substitutions, the presentation of the same epitope in multiple registered has been shown to alter T cell specificity, with particular relevance to type 1 diabetes (60, 61).

Additionally to alterations in the core regions of the peptide, we have demonstrated that antigenic epitopes incorporating PFR modifications can augment T cell activation via enhanced TCR binding to pMHC-II (35). In the present study, we generated multimeric DR1 heterodimers refolded with wild-type or PFR-modified peptides to assess the potential benefits of this approach in an ex vivo setting. Our data show unequivocally that “basic tail” substitutions in the C-terminal PFR can dramatically enhance the avidity of pMHC-II multimers for cognate CD4^+^ T cells. Moreover, such PFR-modified pMHC-II multimers proved superior in head-to-head comparisons with their wild-type counterparts, enabling the reliable detection of Ag-specific CD4^+^ T cells by flow cytometry. These findings offer a generic solution to the problem of “missing clonotypes” that lie below the avidity threshold required for detection with conventional pMHC-II multimers (15). We have previously shown that modifications to PFRs can increase TCR binding in other MHC-II alleles, such as HLA-DR*0401 (35). Because the presentation of peptides by MHC-II molecules seems to be relatively conserved, we are hopeful that PFR modifications could have far-reaching utility to improve the avidity of tetramer reagents in a wide range of MHC-II systems.

To extend these findings, we conducted a detailed ex vivo phenotypic analysis of Ag-specific CD4^+^ T cells expanded by influenza vaccination. The DR1-Flu3 PFR-modified multimer identified a larger population of cognate CD4^+^ T cells compared with the wild-type DR1-Flu1 multimer, enabling a more robust phenotypic evaluation of the vaccine-induced immune response. A marked enrichment of central memory (CD27^+^CD45RO^+^) cells was apparent in the multimer^+^ population relative to the CD4^+^ T cell compartment as a whole. Moreover, these cells were specifically expanded after vaccination. These data demonstrate the practical application of PFR-modified multimers for the ex vivo characterization of Ag-specific CD4^+^ T cells.

Collectively, our results indicate that tailored PFR modifications can overcome intrinsic constraints that limit the utility of wild-type pMHC-II multimers in ex vivo analyses of Ag-specific CD4^+^ T cell populations. Although further work is required to confirm these findings in other systems, we anticipate that our approach will be broadly applicable across Ag specificities and restriction elements, providing a much needed addition to the immunologist’s toolbox.

## Supplementary Material

Data Supplement
