# Supplementary material for: Enhanced Detection of Antigen-Specific CD4+ T Cells Using Altered Peptide Flanking Residue Peptide–MHC Class II Multimers
Source: J Immunol. 2015 Nov 9;195(12):5827–36. doi: 10.4049/jimmunol.1402787 (PMC4671089; doi:10.4049/jimmunol.1402787)
Supplement: Data Supplement [file JI_1402787.zip › JI_1402787_Supplemental_Figures_1.pdf]

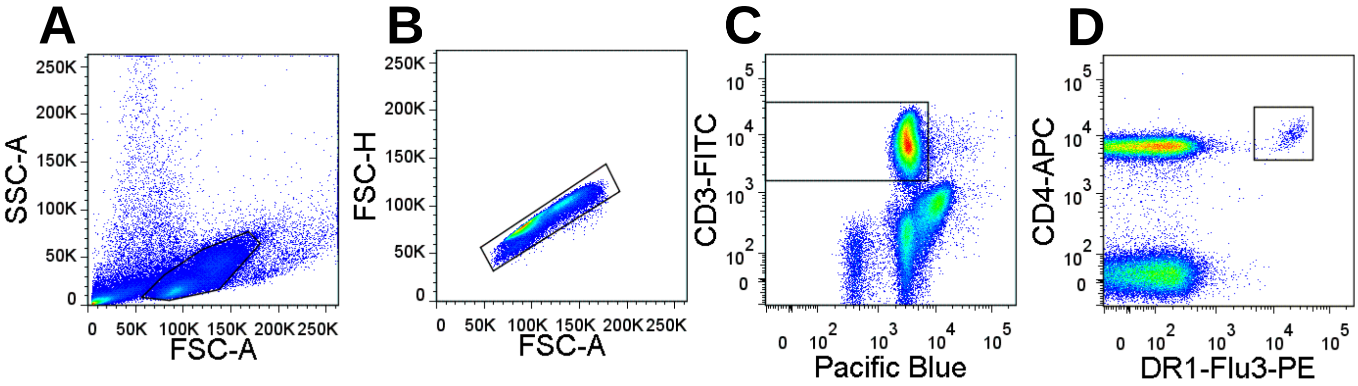

Supplementary Figure S1. Gating strategy used to identify viable CD3^{+}CD4^{+} tetramer-positive events. **(A)** Forward scatter area (FSC-A) *versus* side scatter-area (SSC-A). **(B)** Forward scatter area (FSC-A) *versus* forward scatter height (FSC-H). **(C)** Pacific blue dump channel *versus* CD3- FITC. **(D)** Tetramer-PE *versus* CD4-APC.

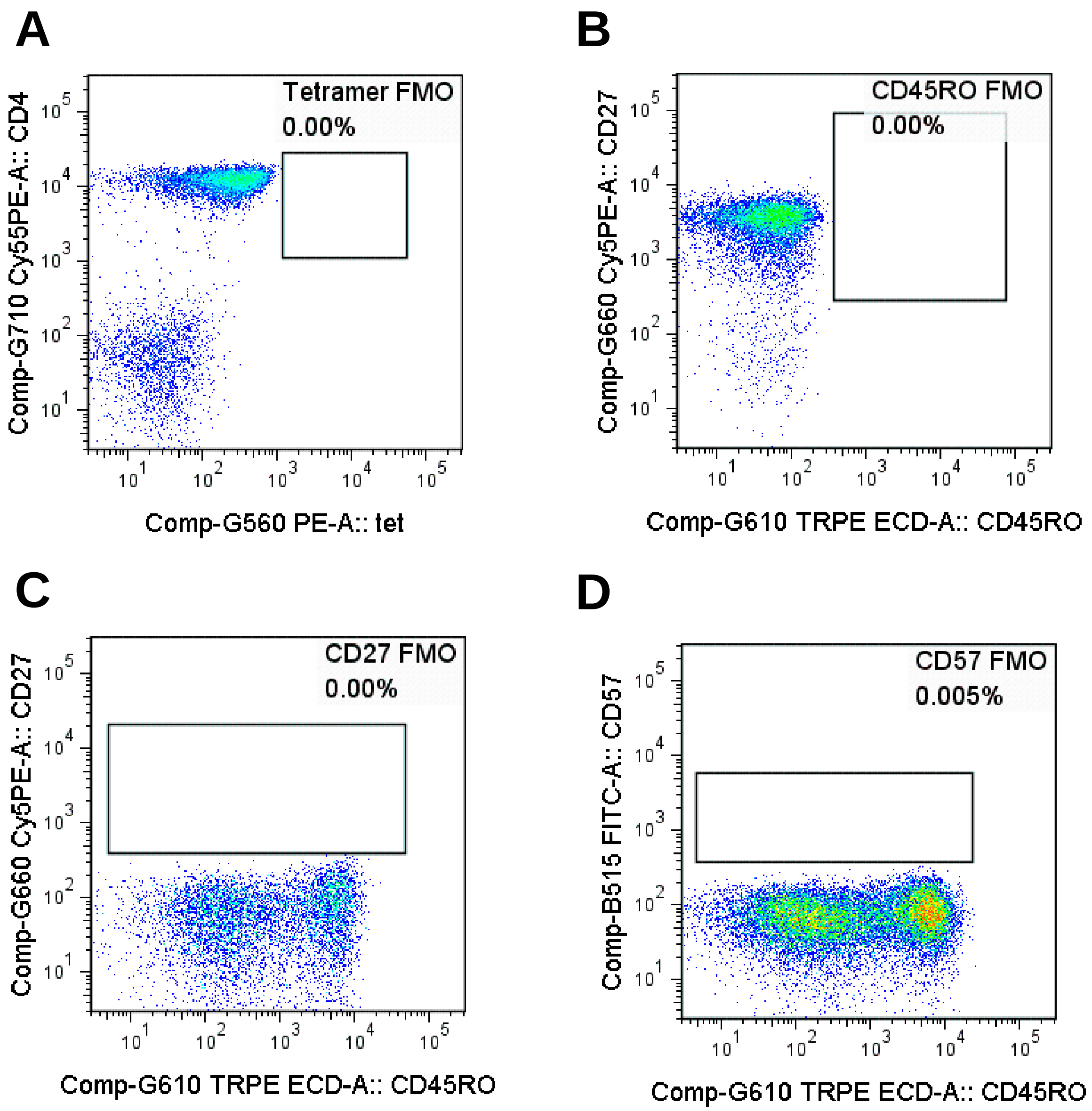

Supplementary Figure S2. ***Ex vivo*** **phenotype analysis: fluorescence minus one (FMO) controls.** To be able to determine where to place the gates during the tetramer and subsequent phenotyping analysis FMO controls were carried out. This was performed by removing individual antibodies from the staining panel. **(A)** Tetramer FMO, FACS plot of CD4 *versus* tetramer. **(B)** CD45RO FMO, FACS plot of CD27 *versus* CD45RO. **(C)** CD27 FMO, FACS plot of CD27 *versus* CD45RO. **(D)** CD57 FMO, FACS plot of CD57 *versus* CD45RO.
